# Exposure to Heavy Ion Radiation Induces Persistent Oxidative Stress in Mouse Intestine

**DOI:** 10.1371/journal.pone.0042224

**Published:** 2012-08-24

**Authors:** Kamal Datta, Shubhankar Suman, Bhaskar V. S. Kallakury, Albert J. Fornace

**Affiliations:** 1 Department of Biochemistry and Molecular and Cell Biology, Georgetown University Medical Center, Lombardi Comprehensive Cancer Center, Washington, D.C., United States of America; 2 Department of Pathology, Georgetown University Medical Center, Washington, D.C., United States of America; 3 Center of Excellence In Genomic Medicine Research (CEGMR), King Abdulaziz University, Jeddah, Saudi Arabia; National Taiwan University, Taiwan

## Abstract

Ionizing radiation-induced oxidative stress is attributed to generation of reactive oxygen species (ROS) due to radiolysis of water molecules and is short lived. Persistent oxidative stress has also been observed after radiation exposure and is implicated in the late effects of radiation. The goal of this study was to determine if long-term oxidative stress in freshly isolated mouse intestinal epithelial cells (IEC) is dependent on radiation quality at a dose relevant to fractionated radiotherapy. Mice (C57BL/6J; 6 to 8 weeks; female) were irradiated with 2 Gy of γ-rays, a low-linear energy transfer (LET) radiation, and intestinal tissues and IEC were collected 1 year after radiation exposure. Intracellular ROS, mitochondrial function, and antioxidant activity in IEC were studied by flow cytometry and biochemical assays. Oxidative DNA damage, cell death, and mitogenic activity in IEC were assessed by immunohistochemistry. Effects of γ radiation were compared to ^56^Fe radiation (iso-toxic dose: 1.6 Gy; energy: 1000 MeV/nucleon; LET: 148 keV/µm), we used as representative of high-LET radiation, since it's one of the important sources of high Z and high energy (HZE) radiation in cosmic rays. Radiation quality affected the level of persistent oxidative stress with higher elevation of intracellular ROS and mitochondrial superoxide in high-LET ^56^Fe radiation compared to unirradiated controls and γ radiation. NADPH oxidase activity, mitochondrial membrane damage, and loss of mitochondrial membrane potential were greater in ^56^Fe-irradiated mice. Compared to γ radiation oxidative DNA damage was higher, cell death ratio was unchanged, and mitotic activity was increased after ^56^Fe radiation. Taken together our results indicate that long-term functional dysregulation of mitochondria and increased NADPH oxidase activity are major contributing factors towards heavy ion radiation-induced persistent oxidative stress in IEC with potential for neoplastic transformation.

## Introduction

Qualitatively low-linear energy transfer (LET) radiation like γ-rays and x-rays are sparsely ionizing. In contrast, high-LET radiation like α-particles, neutrons, and heavy ions are densely ionizing and are much more damaging to tissues and cells than low-LET radiation [1]–[3]. High-LET radiation from high energy charged (HZE) particles like ^56^Fe, ^28^Si, and ^12^C contributes substantially to the dose equivalent of the cosmic radiation [4] and it is estimated that during a 3-year long space mission significant number of cells in the body will be exposed to high-LET heavy ion radiation [1]. Considering its high relative biological effectiveness (RBE) or quality factor and differential physical characteristics [5], [6], high-LET radiation exposure poses greater health risk than low-LET radiation to astronauts and is a major concern for long duration space missions [1], [7]–[11]. With increasing interest in space tourism, cosmic radiation exposure is also a risk factor for civilian population [12].

Colorectal cancer (CRC) is not only frequent but is also the second leading cause of cancer mortality in the Unites States [13] with an estimated 143,460 new cases of the disease in the US in 2012. Studies in atomic bomb survivors and in other epidemiological studies implicate radiation as a risk factor for CRC [14]–[16]. However, unlike low-LET radiation, CRC risk prediction of high-LET radiation is problematic due to the paucity of *in vivo* long-term follow up data in human or animal models [1], [7]. Previously, we have shown that exposure to high-LET ^56^Fe radiation, relative to low-LET exposure, resulted in higher intestinal tumorigenesis in APC^Min/+^ mice [17], a well-studied CRC animal model developing adenomas of intestinal epithelial cells (IEC). However, we are yet to clearly understand how high-LET radiation differentially altered intestinal epithelial cell homeostasis to promote greater tumorigenesis. Acquiring *in vivo* data will allow us to develop risk models of high-LET radiation exposure not only to lessen the uncertainty of health risks of space travel but also to develop strategies to protect healthy tissues from other sources of high-LET radiation such as environmental radon and radiotherapeutic heavy ion radiation exposures [18], [19].

Delayed effects of radiation exposure have been attributed to persistent oxidative stress in tissues as a result of functional changes in mitochondria, a major source of reactive oxygen species (ROS) in cells [20]–[24]. Although ROS are generated in mitochondria as a part of normal oxidative metabolism, enhanced ROS production could occur following damage to mitochondrial membrane and loss of mitochondrial membrane potential (MMP) as result of exposure to stressors like radiation and chemicals. Indeed, low-LET radiation exposure has been linked to mitochondrial membrane damage and alterations in MMP promoting ROS generation [25], [26]. NADPH oxidase, another important source of ROS, is activated by radiation exposure in murine hematopoietic stem cells leading to persistent oxidative stress and genomic instability [27], [28]. Isoforms of NADPH oxidase, NOX1 and NOX2, are expressed in intestine [29], [30] and if activated would enhance ROS production in IEC. Increased ROS production in IEC is not only linked to activation of inflammatory pathways and initiation and promotion of cancer but it is also associated with inflammatory bowel diseases and radiation damage to gastrointestinal (GI) tract [31]–[33]. However, radiation-induced delayed oxidative stress in relation to radiation quality has not been characterized *in vivo* in IEC.

Antioxidant enzyme Cu-Zn superoxide dismutase (SOD1) protects host cells from superoxide present outside mitochondria, Mn-superoxide dismutase (SOD2) neutralizes superoxide in mitochondria, and catalase neutralizes hydrogen peroxide generated from the dismutation of superoxide. Disbalance between ROS generation and antioxidant activity results in oxidative stress. Increased ROS induces oxidative DNA damage of which 8-oxo-dG is a known marker and at sub-lethal level increased ROS could act as a second messenger in cellular signaling pathways to trigger proliferative signals thus increasing the chance of passing the genomic alterations into the progeny [33]–[35]. Increased ROS is also known to produce DNA double strand break (DSB) and persistently increased ROS could result is sustained DSB production and perpetual DNA damage response (DDR) signaling with chronic accumulation of known markers such as 53BP1 and γH2AX. While high-LET radiation has been shown to induce higher oxidative stress [36] and greater DNA damage response determined by 53BP1 and γH2AX accumulation [37]–[40] in cells than low-LET radiation, most of these studies were short-term and *in vitro* and none have investigated these markers *in vivo* in IEC. Here we report that high-LET radiation induced persistent oxidative stress in IEC one year after exposure. Our results suggest that sustained oxidative stress is mediated by destabilized and damaged mitochondria as well as by increased NADPH oxidase activity. Persistent oxidative stress in IEC was further enhanced by reduced activity of antioxidant enzymes in high-LET radiation exposed animals. We also show greater oxidative DNA damage, higher mitotic activity, and more 53BP1 foci accumulation in IEC after high-LET irradiation relative to γ radiation.

## Materials and Methods

### Animals

The source of the mice (C57BL/6J, female, 6 to 8 weeks) used in this study was Jackson Laboratories (Bar Harbor, ME, USA). Mice were exposed to ^56^Fe radiation at the NASA Space Radiation Laboratory (NSRL) located at the Brookhaven National Laboratory (BNL). Mice were housed at the BNL animal facility and on the day after irradiation mice were shipped from BNL early in the morning and brought to the Georgetown University (GU) animal facility in the afternoon in a temperature-controlled environment along with the respective sham irradiated control groups. A ^137^Cs source was used for γ radiation and the corresponding control group was sham irradiated. All animal procedures were performed in accordance with the protocols approved by the GU and BNL Institutional Animal Care and Use Committee (IACUC) and mice were provided certified rodent diet along with filtered water *ad libitum*. Furthermore, we used the Guide for the Care and Use of Laboratory Animals, prepared by the Institute of Laboratory Animal Resources, National Research Council, and U.S. National Academy of Sciences as guidelines for our research.

### Irradiations

Mice were placed in rectangular clear lucite boxes (3″×1.5″×1.5″) with multiple holes for respiration and exposed to ^56^Fe (energy: 1000 MeV/nucleon; LET: 148 keV/µm), and γ radiation at a dose rate of 1 Gy/min. Mice (n = 6 per experimental group) were exposed to 2 Gy of γ radiation and 1.6 Gy of ^56^Fe radiation and irradiation was repeated in a second experiment with the same number of mice in each group. The dose of ^56^Fe radiation was iso-toxic to the γ radiation dose and was calculated using a quality factor (RBE) of 1.25 determined earlier [8].

### Harvesting intestinal tissue and isolation of intestinal epithelial cells (IEC)

One year after radiation exposure mice were euthanized as per approved protocol and small intestine was dissected, intestinal lumen was flushed with phosphate buffered saline (PBS) at room temperature, and jejunal region was used for isolation of IEC. For histology 3 cm of jejunum was fixed in 10% buffered formalin, paraffin embedded, and 4 µm sections were made for immunohistochemistry. For IEC, intestinal lumen was inverted and cells were isolated according to the protocol described earlier [41], [42] with some modification. Briefly, inverted intestinal lumen was immersed in 2 ml of Solution-A (27 mM Sodium citrate, 1.5 mM KCl, 96 mM NaCl, 8 mM KH_2_PO_4_ and 5.6 mM NaH_2_PO_4_ at pH 7.3) for 15 min followed by 30 min incubation in 1.5 ml of Solution-B on a shaker plate (1.5 mM EDTA, 0.5 mM dithiothreitol, 25 ng/ml amphotericin-B, 100 U/ml penicillin, 100 µg/ml streptomycin, and 1% gentamycin in PBS). Detached cells were made into single cell suspension by repeated pipetting and passed through a 70-micron mesh (BD Biosciences, Bedford, MA) to get a homogenous single cell suspension. Cells were pelleted by centrifugation (900×g for 5 min at room temperature), re-suspended in Medium 199 (Cat# 12-119, Lonza, Wakersville, MD) with antibiotics (25 ng/ml amphotericin-B, 100 U/ml penicillin, 100 µg/ml streptomycin and 1% gentamycin) and allowed to remain in media overnight in 6-well plates to recover from isolation stress. IEC obtained by this procedure includes cells from both the villi and crypts and was characterized by morphological observation under phase contrast microscope and by alkaline phosphatase assay (data not shown) as described previously [41], [42] and cell viability was checked by trypan blue (>97% viability). Cells isolated from each mouse were processed separately and used for flow cytometry and western blot. While for flow cytometry samples from 6 mice in each group were processed in triplicate for each experiment, for western blot samples from 6 mice in each group were pooled to prepare the cell lysate. Also, IEC lysates from 6 mice were used for activity assays and nitrate/nitrite estimation.

### Intracellular ROS measurements

Intracellular ROS level in IEC was measured using the fluorescent probe 2′7′-dicholorodihydrofluorescein di-acetate (H_2_DCFDA, Invitrogen, Carlsbad, CA) as described previously [43]. Briefly, cells were pelleted (900×g for 5 min), media discarded, and cells washed once with PBS. Cells were resuspended in 1 ml of PBS, incubated in the dark with the fluorescent probe (final concentration 10 µM) at 37°C for 20 minutes, and were washed in PBS once. Cells were resuspended in 500 µl of PBS and data acquired by flow cytometry (BD Biosciences, Bedford, MA).

### Mitochondrial superoxide assay

Mitochondrial superoxide level in IEC was measured using a specific mitochondrial superoxide indicator, MitoSOX red (Invitrogen) as per manufacturer's instructions. Briefly, cells were incubated in dark with MitoSOX red in PBS (5 µM; 20 min at 37°C). Cells were washed with PBS, resuspended in PBS (500 µl) and data acquired by flow cytometry (BD Biosciences).

### Mitochondrial membrane potential (MMP) analysis

Changes in MMP were assessed in IEC as per protocol described previously [44]. Briefly, cells were pelleted (900×g for 5 min), media discarded, and pellet resuspended in 1 µM Rhodamine 123 (Invitrogen) in buffer containing 10 mM HEPES, 145 mM NaCl, 5 mM KCl, 1.0 mM MgCl_2_-6H_2_O, 1.8 mM CaCl_2_-2H_2_O, pH 7.4. Cells were incubated in dark for 30 min at 37°C. Cells were washed once with PBS and resuspended in 500 µl of PBS. Data acquired by flow cytometry (BD Biosciences).

### Measuring cardiolipin content of mitochondrial membrane

In eukaryotic cells cardiolipin, a phospholipid, is associated only with the mitochondrial inner membrane and oxidation of cardiolipin is an indicator of mitochondrial membrane damage [45], [46]. The fluorescent probe nonyl acridine orange (NAO; Invitrogen) does not bind to the oxidized cardiolipin and quantification of cardiolipin content is a measure of oxidative damage to mitochondrial membrane [45]. Cardiolipin content of mitochondrial membrane in IEC was measured using a protocol described before [46]. Briefly, cells were incubated for 30 min at 37°C in dark with 50 nanomolar NAO in a buffer containing NaCl 156 mM; KCl 3 mM; MgSO_4_ 2 mM; KH_2_PO_4_ 1.25 mM; CaCl_2_ 2 mM; D-glucose 10 mM; HEPES 10 mM; pH adjusted to 7.35. Cells were washed in PBS, re-suspended in 500 µl PBS and data acquired by flow cytometry (BD Biosciences).

### SOD1 and SOD2 activity assay

SOD1 and SOD2 activity in IEC was assessed using a kit based assay as per manufacturer's instructions (Cat#706002, Caymen Chemicals, Ann Arbor, MI). Briefly, cells were washed with ice cold PBS and homogenized in ice cold lysis buffer (20 mM HEPES, pH 7.2, containing 1 mM EDTA, 210 mM mannitol, and 70 mM sucrose). Cell lysate was centrifuged at 1500×g for 5 min at 4°C and supernatant collected. Mitochondrial pellet was obtained by further centrifuging the supernatant at 10,000×g for 15 min at 4°C. The supernatant fraction from this centrifuge was assayed for cytosolic SOD (SOD1). The mitochondrial pellet was re-suspended in cold lysis buffer and homogenized by sonication and was assayed for mitochondrial SOD (SOD2). Absorbance of both the assays was read at 450 nm and results were calculated as unit/mg of protein where one unit of SOD is the amount of enzyme needed to exhibit 50% dismutation of the superoxide radicals (Cayman Chemicals). Protein estimation was done using Bradford's protein assay as per procedure standardized in the laboratory.

### Catalase activity assay

Catalase activity in IEC was assayed using a commercially available kit (Cat#707002, Cayman Chemicals) as per manufacturer's instructions. Briefly, cells were washed with cold PBS and homogenized in cold lysis buffer (50 mM potassium phosphate, pH 7.0, containing 1 mM EDTA). The lysate was centrifuged at 10,000×g for 15 min at 4°C. Supernatant was collected for catalase assay. Absorbance was measured at 540 nm and results expressed as nmol/min/mg of protein. The assay considers one unit as the amount of catalase needed to form 1.0 nmol of formaldehyde per minute at 25°C (Cayman Chemicals). Protein estimation was performed using Bradford's reagent.

### NADPH oxidase activity assay

NADPH oxidase activity in IEC was assessed using a luminescence-based assay as per protocol described earlier with modifications [47]. Briefly, 10 µl of cell homogenate was added to the 90 µl of reaction mix (50 mM phosphate buffer (pH 7.0) containing 1 mM EGTA, 150 mM Sucrose, 500 µM lucigenin and 100 µM NADPH). The assay is based on the ability of lucigenin to accept electrons produced by NADPH oxidase activity. The lucigenin luminescence is directly proportional to electrons generated by NADPH oxidase and higher luminescence is indicative of higher NADPH oxidase activity. Luminescence was recorded at 0 min and at 15 min. Change in luminescence was calculated by subtracting initial luminescence from the final luminescence and data presented as relative light unit (RLU)/µg of protein.

### Lipid peroxidation assay

Lipid peroxidation was detected using a specific fluorescent indicator, cis-parinaric acid (Invitrogen) as per protocol described before [48]. Briefly, cells were loaded with 5 µM cis-parinaric acid and incubated for 60 min at 37°C in dark followed by a PBS wash. Fluorescence was measured using 320 nm excitation and 420 nm emission. Decrease in cis-parinaric acid fluorescence indicated lipid peroxidation and results are presented as percent change in fluorescent intensity.

### Cellular nitrate/nitrite estimation

Nitrosative stress marker nitrate/nitrite was measured in IEC using commercially available kit as per manufacturer's instructions (Cat # 760871, Cayman Chemicals). Briefly, cells were washed with PBS and the cell pellet was homogenized in PBS (pH 7.4), centrifuged at 10,000×g for 60 min, and samples were processed as per instructions in the kit. Supernatant was assayed for nitrate/nitrite using Griess reagent. Absorbance was acquired at 550 nm using a 96-well plate reader (M^e2^, Molecular Devices, Sunnyvale, CA). Nitrate/nitrite concentration in test samples measured in triplicate was calculated using a standard curve and results expressed in µM.

### Immunohistochemistry in intestinal sections

Immunohistochemical staining for 8-oxo-dG was performed using primary antibody from Trevigen (Gaithersburg, MD) as per manufacturer's instruction with modifications. Briefly, depraffinized sections were incubated overnight with anti-8-oxo-dG at 4°C. After necessary washing steps, sections were developed using horseradish peroxidase (HRP) conjugated secondary antibody and diaminobenzidine (DAB) detection system as described previously [49]. For phospho-histone H3 (p-H3, ser-10) immunostaining was performed using primary antibody from Millipore (Billerica, MA) as per protocol described previously [50]. Briefly, sections were depraffinized and antigen retrieval was performed in pH 6.0 citrate buffer (Dako, Carpinteria, CA). After quenching endogenous peroxidase activity and incubation in blocking buffer (0.1% bovine serum albumin in PBS), the sections were exposed to p-H3 antibody (dilution 1∶100) for 1.5 hr. Signal detection and color development was performed using SuperPicture™ 3^rd^ Gen IHC detection kit (Cat# 87-9673; Invitrogen). Slides were mounted and visualized under bright field microscopy and quantification was performed at 20× magnification using ImageJ v1.45 software as described previously [51]. Representative images captured at 20× magnification are presented in results. For immunostaining of 53BP1, following the blocking step described for p-H3, intestinal sections were incubated overnight with anti-53BP1 antibody (dilution 1∶200, PA116566, Thermo Fisher Scientific, Rockford, IL) at 4°C. After necessary washing steps sections were incubated with AlexaFluor488-conjugated goat-anti-rabbit antibody (A-11034, Life Technologies, Grand Island, NY) for 1 h in dark at room temperature. Samples were washed and mounted using DAPI containing VECTASHIELD mounting medium (H-1200, Vector Laboratories, Burlingame, CA). Sections were visualized and images captured at 60× magnification in oil using an Olympus BX61 DSU fluorescent microscope and images were analyzed using SlideBook v5.0 software (Intelligent Imaging Innovations, Inc, Denver, CO).

### Terminal deoxynucleotidyl transferase dUTP nick end labeling (TUNEL) assay

TUNEL assay was performed on intestinal sections using ApopTag plus peroxidase *in situ* apoptosis detection kit (Cat#S7101, Millipore, Billerica, MA) as per manufacturer's instruction. Briefly, sections were depraffinized, pretreated with proteinase K, and endogenous peroxidase was quenched using H_2_O_2_. The sections were incubated with deoxynucleotidal transferase (TdT), developed with diaminobenzidine (DAB) detection system, and counterstained with hematoxylin. Stained sections were visualized under bright field microscope and images were captured at 20× microscopic magnification for quantification. Representative images captured at 20× magnification are presented in results.

### Data analysis and statistics

Flow cytometry data were analyzed by WinMDI v2.9 and average percent change of mean fluorescence from triplicate samples of 6 mice are plotted graphically and a representative histogram comparing untreated control to treated samples in one animal is shown in the results. Images acquired from 8-oxo-dG, p-H3, and TUNEL staining were analyzed using color deconvolution and Image-based Tool for Counting Nuclei (ITCN) plug-ins of ImageJ v1.45 software by two observers blinded to treatment groups as per protocol described earlier [51], [52]. We used 24 random image frames (4 frames per section; n = 6 per group) captured at 20× microscopic magnification for analysis and mean data from one experiment are presented graphically as average number of positive nuclei per 20× field and a representative image (20× magnification) from one animal of each group of one experiment is shown in the results. Data in figures are presented as mean ± standard error of mean (SEM). An independent viewer counted 53BP1 foci by observation in 24 random image frames (4 frames per section; n = 6 per group) captured at 60× magnification and results are expressed as average foci per high power field (HPF) and a representative image (60× magnification) from one animal of each group of one experiment is shown in the results. Difference between the groups was analyzed using two-tailed paired student's *t* test. Results were considered significant at p<0.05.

## Results

### Persistent oxidative stress and lipid peroxidation in IEC is dependent on radiation quality

**Figure 1:**
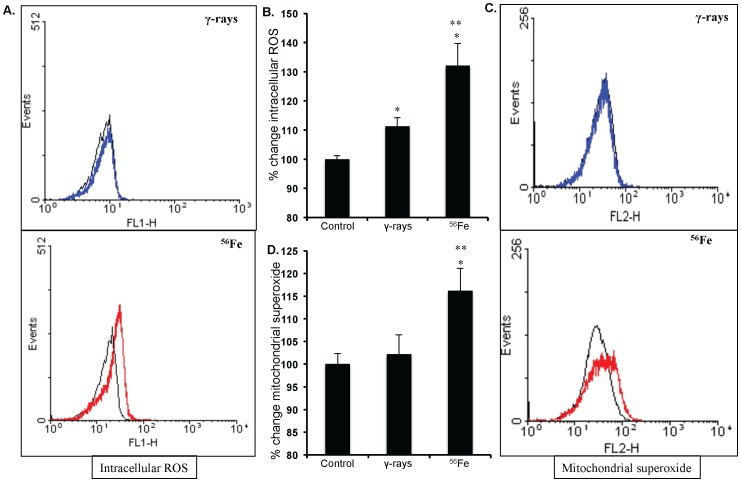
Measuring ROS and mitochondrial superoxide in IEC. A) Flow cytometry histogram showing change in ROS level after exposure to γ, and ^56^Fe radiation. B) Quantification of ROS level presented as percent change of mean fluorescence. C) Flow cytometry histogram showing mitochondrial superoxide levels after exposure to γ and ^56^Fe radiation. D) Quantification of mitochondrial superoxide level presented as percent change of mean fluorescence. Cells from unirradiated mice were used as controls. *significant compared to control; **significant compared to γ radiation. Histogram colors - Black: control, Blue: γ radiation, and Red: ^56^Fe radiation samples.

**Figure 2:**
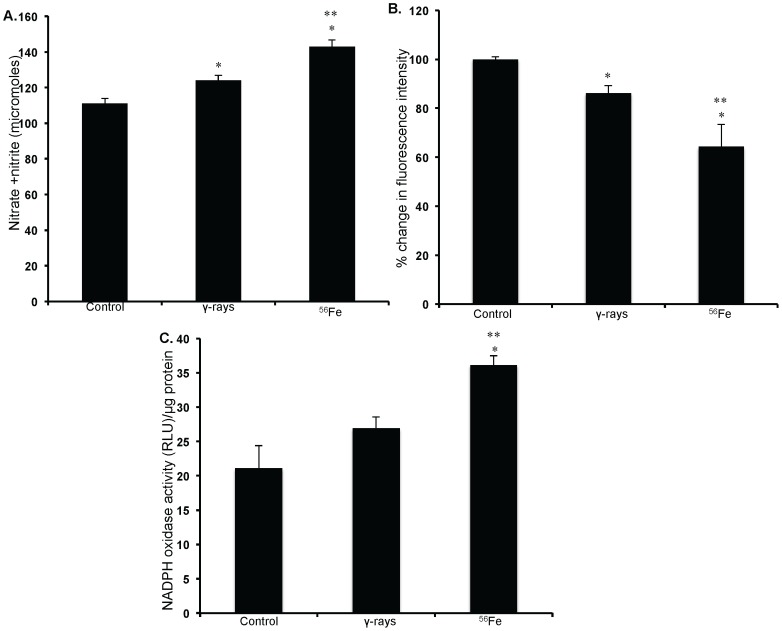
Measurement of oxidative stress in IEC. A) Nitrate/nitrite levels were measured using Griess reagent in IEC after radiation exposure. B) Lipid peroxidation assessed in IEC using cis-parnaric acid is presented as mean fluorescent intensity. Decrease of cis-parnaric acid fluorescence is proportionate to increase in lipid peroxidation. C) NADPH oxidase activity was determined in IEC by measuring lucigenin luminescence after radiation exposure and is presented as relative light unit (RLU) per µg protein. Change in lucigenin luminescence is proportional to change in NADPH oxidase activity. *significant compared to control; **significant compared to γ radiation.

Our observations show that compared to unirradiated control levels of ROS increased significantly 1 year after γ as well as in ^56^Fe radiation (p<0.02 for γ and p<0.01 for ^56^Fe; **Figure 1A and B**). Importantly, however, increase was significantly more in high-LET ^56^Fe radiation than in low-LET γ radiation (p<0.04 compared to γ radiation; **Figure 1B**). Mitochondrial superoxide production was also significantly more in ^56^Fe compared to γ radiation (p<0.001 for ^56^Fe compared to γ radiation; **Figure 1C and D**). Nitrate/nitrite levels in cell lysate indicating nitrosative stress in IEC were more in both the γ and the ^56^Fe radiation (compared to control p<0.02 for γ, and p<0.007 for ^56^Fe radiation; **Figure 2A**). However, nitrate/nitrite levels compared to γ radiation were significantly higher in ^56^Fe-irradiated samples (p<0.04 for ^56^Fe radiation; **Figure 2A**). Lipid peroxidation in IEC measured by decrease in cis-parinaric acid fluorescence was significantly more in ^56^Fe radiation (p<0.05 for ^56^Fe compared to γ radiation; **Figure 2B**). Persistent lipid peroxidation, less than ^56^Fe radiation, was also observed after γ radiation (p<0.002 for γ compared to control).

### Changes in NADPH oxidase activity and mitochondrial physiology contribute to radiation quality dependent oxidative stress in IEC

**Figure 3:**
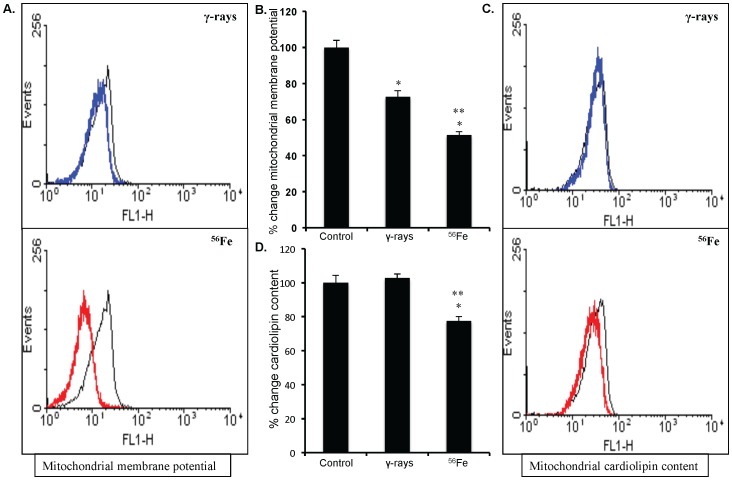
Assessing mitochondrial membrane potential (MMP) and cardiolipin content in IEC. A) Flow cytometry histogram showing changes in MMP after exposure to γ, and ^56^Fe radiation. B) Quantification of MMP change presented as percent change of mean fluorescence. C) Flow cytometry histogram showing changes in mitochondrial cardiolipin content after exposure to γ, and ^56^Fe radiation. D) Mean fluorescence intensity calculated from flow cytometry histograms are presented as percent change of mean fluorescence. Cells from unirradiated mice were used as controls. Histogram color - Black: control, Blue: γ radiation, and Red: ^56^Fe radiation samples. *significant compared to control; **significant compared to γ radiation.

Compared to γ radiation NADPH oxidase activity was significantly more in cell lysate from ^56^Fe-irradiated samples (p<0.01; **Figure 2C**). The MMP compared to control was significantly reduced in all radiation types (p<0.0004 for γ, and p<0.0008 for ^56^Fe radiation). However, a radiation quality dependent effect was observed in MMP loss and compared to γ radiation significantly greater loss of MMP was observed after ^56^Fe radiation (p<0.001 compared to γ; **Figure 3A and B**). In addition to change in MMP, we also assessed changes in mitochondrial cardiolipin content, an indicator of mitochondrial membrane damage. There was no significant change in mitochondrial cardiolipin after γ radiation. However, a significant decrease in cardiolipin content was observed after ^56^Fe radiation (compared to γ radiation p<0.001 for ^56^Fe; **Figure 3C and D**).

### Lowering of antioxidant enzyme activity was more after high-LET irradiation

**Figure 4:**
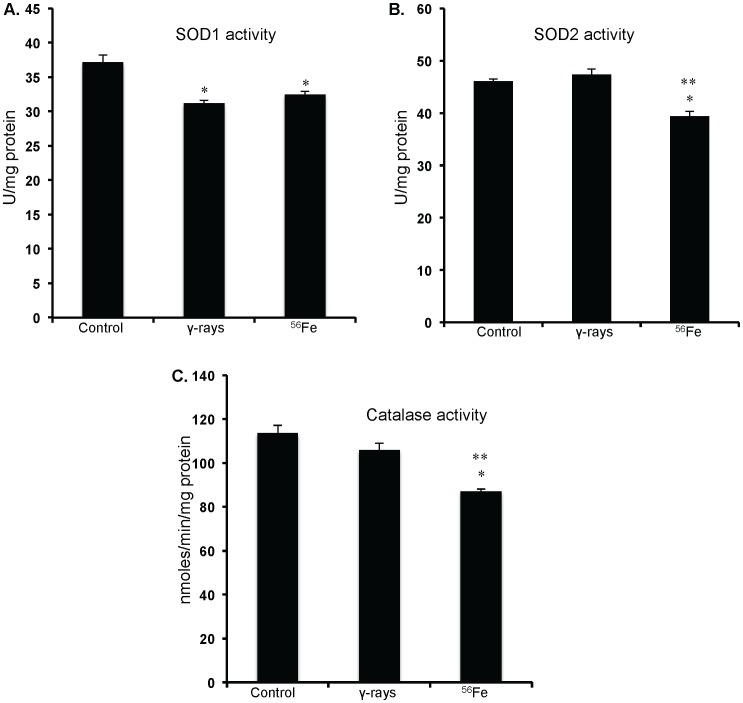
Antioxidant enzyme activity in IEC. A) SOD1 activity after exposure to γ and ^56^Fe radiation. B) SOD2 activity after exposure to γ and ^56^Fe radiation. C) Catalase activity after exposure to γ and ^56^Fe radiation. *significant compared to control; **significant compared to γ radiation.

Both cytosolic and mitochondrial superoxide dismutase (SOD1 and 2) activity is significantly reduced in ^56^Fe treated groups (compared to control p<0.02 for SOD1 and for SOD2; **Figure 4A and B**). SOD2 was also significantly lower in ^56^Fe than in γ radiation (p<0.01; **Figure 4B**). Compared to control there was no significant alterations in the SOD2 activity after γ radiation (**Figure 4B**). However, lowering of the SOD1 activity in γ radiation was similar to ^56^Fe radiation (**Figure 4A**). Furthermore, compared to control and γ radiation a significant decrease in catalase activity was observed after ^56^Fe radiation (compared to control p<0.02 and compared to γ radiation p<0.03; **Figure 4C**).

### Increased oxidative DNA damage, persistent DNA damage response and increased mitogenic activity in intestinal cells

**Figure 5:**
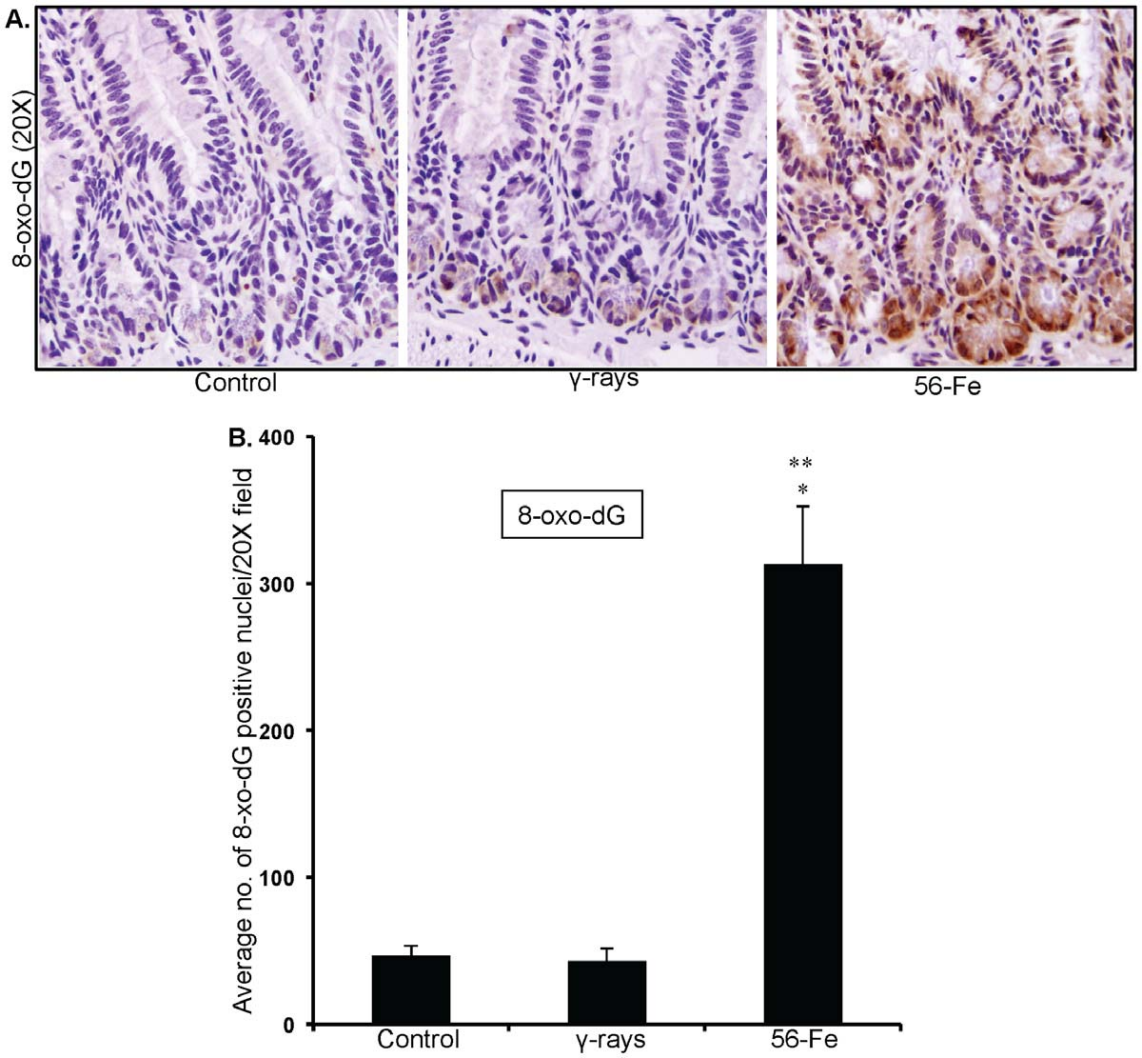
Higher oxidative DNA damage after ^56^Fe radiation. A) Immunohistochemical staining of intestinal sections for 8-oxo-dG after exposure to γ and ^56^Fe radiation. B) Quantification of 8-oxo-dG staining in intestinal sections. *significant compared to control; **significant compared to γ radiation.

**Figure 6:**
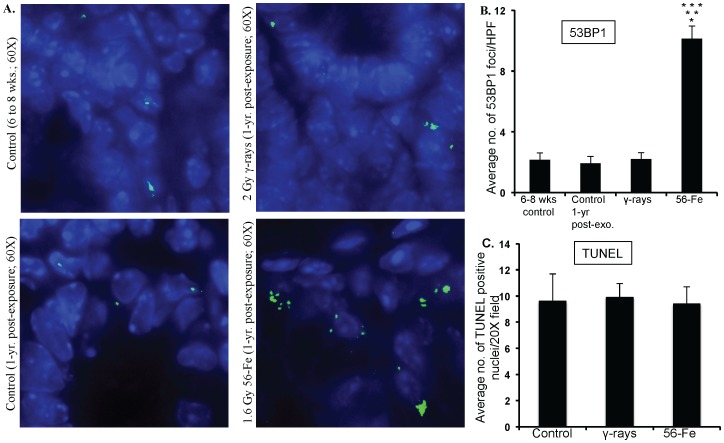
Assessing 53BP1 foci and cell death in intestinal sections after γ and ^56^Fe radiation exposure. A) Intestinal sections from 6 to 8 week control, 1-year post-exposure control, 2 Gy γ, and 1.6 Gy ^56^Fe 1-year post-exposure mice were immunofluorescently stained for 53BP1. B) Quantification of 53BP1 foci in intestinal cell nuclei counterstained with DAPI. C) Quantification of TUNEL staining of intestinal sections after exposure to γ and ^56^Fe radiation. *significant compared to 6–8 wks unirradiated control; **significant compared to 1-year post-exposure unirradiated control; ***significant compared to γ radiation.

**Figure 7:**
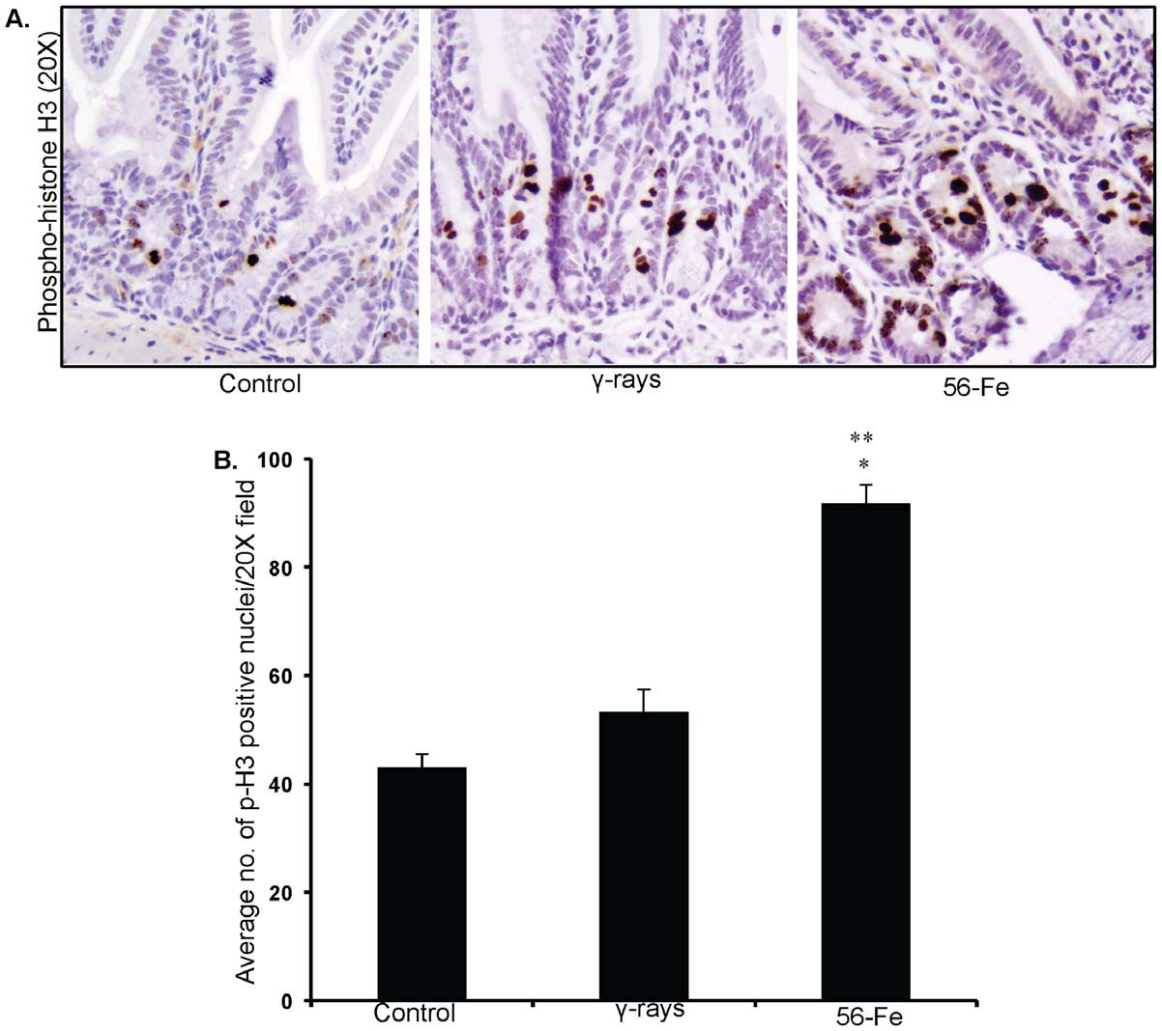
Increased mitogenic activity in intestinal sections after ^56^Fe radiation. A) Immunohistochemical staining of intestinal sections for phospho histone H3 (p-H3) after γ and ^56^Fe radiation. B) Quantification of p-H3 positive nuclei in intestinal cells. *significant compared to control; **significant compared to γ radiation.

Effects of persistent oxidative stress were assessed in intestinal sections by immunohistochemistry. Our observations of 8-oxo-dG staining demonstrate that compared to control and γ radiation there was markedly higher staining in ^56^Fe-irradiated samples (**Figure 5A**). Quantification of 8-oxo-dG showed significantly more oxidative damage to DNA in ^56^Fe samples (compared to control and γ radiation p<0.0001; **Figure 5B**). Furthermore, 53BP1 foci indicative of DNA double strand breaks and DNA damage response were distinctly more in ^56^Fe irradiated mice (**Figure 6A**). Quantification of foci showed significantly higher 53BP1 accumulation after ^56^Fe irradiation relative to two unirradiated control groups (6–8 week old young mice and 1-year post-exposure older mice) and γ radiation (compared to controls and γ radiation p<0.0002; **Figure 6B**). Both 8-oxo-dG and 53BP1 staining levels in young (6–8 wks) and older (1-year post-exposure) control mice were similar (data not shown and **Figure 6B**). Subsequently, we were interested to find if DNA damage were causing cell death in intestinal cells. Surprisingly, we did not observe compared to control any significant increase in TUNEL positive staining in γ as well as in ^56^Fe irradiated samples (**Figure 6C**). Because there were increase oxidative DNA damage but no alterations in cell death, we were interested to determine whether intestinal cells were dividing or have they undergone replicative senescence. Our p-H3 immunostaining indicating mitosis showed significantly higher staining in ^56^Fe irradiated samples (compared to control p<0.00009 and compared to γ radiation p<0.002 for ^56^Fe; **Figure 7A and B**). The p-H3 staining in γ radiation samples was slightly higher but was not significantly different than control (**Figure 7B**).

## Discussion

Much uncertainty exists about long-term health risk from high-LET radiation exposure mostly due to lack of sufficient *in vivo* data [1], [53], [54]. Specifically, we do not have reliable risk estimates of CRC from cosmic radiation exposure and a limiting factor is our scarce understanding of underlying molecular events involving IEC after heavy ion radiation exposure. Here we demonstrate that high-LET radiation induced higher ROS levels *in vivo* in mouse IEC than low-LET radiation even one year after exposure. Our results also indicate that persistent ROS increase correlates with destabilization of mitochondria and activation of NADPH oxidase and this is a likely cause for the ROS effect. Persistent oxidative stress is associated with low-LET radiation induced delayed effects in tissues and cells [21], [23], [24], [55] and our observations of chronic increase in ROS *in vivo* in IEC after low-LET radiation exposure is consistent with published *in vitro* data in other cell types at shorter time points [24], [56]. However, we show for the first time that persistent oxidative stress in mouse intestine is radiation quality dependent with increased potential for long-term more deleterious effects by high-LET radiation. Higher nitrosative stress and lipid peroxidation observed in our study after high-LET radiation is indicative of high levels of ROS reacting with nitric oxide and lipid molecules leading to further increase of oxidants in cells. Importantly, our results support the notion that perturbation of mitochondria and activation of NADPH oxidase, the two major sources of ROS is a prerequisite for this long-term oxidative stress in cells. Taken together we show that the high-LET heavy ion radiation caused greater changes in ROS generating machinery including greater damage to mitochondrial membrane leading to higher oxidative stress than low-LET radiation.

High levels of ROS are known to induce an apoptotic response but sustained sub-lethal oxidative stress has the potential to produce persistent modification of biomolecules including proteins, DNA, and lipids leading to alterations in their functions with adverse cellular consequences. Indeed, DNA remains an important target of ROS mediated damage and sub-lethal persistent oxidative stress induce a number of DNA modifications which may enhance the chances of genomic alterations such as mutations and deletions with harmful consequences especially in tissues like intestine with constantly dividing cells. Our results show that compared to low-LET radiation high-LET radiation caused marked elevation of 8-oxo-dG in intestinal cells. The adduct 8-oxo-dG is an oxidative DNA modification forms as a result of hydroxyl radicals mediated hydroxylation of deoxyguanosine molecule at the C-8 position and is a known marker of oxidative DNA damage [33], [34]. Furthermore, the 8-oxo-dG modifications in DNA have the capability of G:C to T:A transversion which can lead to site-specific heritable mutations in genes of signaling pathways commonly altered in human cancers [33]–[35]. Apart from modifications in DNA, ROS is also capable of inducing DNA breaks including DSBs. If remains unrepaired DSBs could induce cell death, and misrepaired DSBs are known to cause genomic instability, a hallmark of cancer initiation and promotion. Chronic ROS elevation and consequent persistence of DNA damage after ^56^Fe exposure is expected to invoke continued DNA damage response, which in our study is evident by increased accumulation of 53BP1 foci in IEC [57], [58]. Increased accumulation of 53BP1 foci is also indicative of chronic DSB production, which we show to be radiation quality dependent. Importantly, existence of unresolved DSB along with persistent DSB generation opens the possibilities of misrepair and genomic instability, a neoplastic precursor event [2]. Increased ROS is also known to damage proteins and alter their functions. Oxidative stress has been reported to lower antioxidant enzyme activity, which was observed in our study, due to a multitude of factors including decrease translation, changes in post-translational modification, alterations in cell signaling molecules regulating the enzymes, or overwhelming of the antioxidant system [59], [60]. In addition, SOD2 has been reported to exhibit decreased enzyme activity in cells exposed to radiation, and mitochondria have been suggested to be a major contributor to persistent oxidative stress thus corroborating our results [24].

While higher-level ROS is known to induce apoptotic responses, sub-lethal oxidative stress can induce either cellular senescence or proliferation [33]–[35], [61], [62]. Our TUNEL assay results showing a similar cell death pattern in control and irradiated samples led us to conclude that ROS in these cells are sub-lethal and raised the question of senescence vs. proliferation. Exposure of cells to stress is known to activate mitogenic signaling pathways like EGFR/RAS/PI3K-MAPK pathway, which in turn stimulates proliferation and progression into mitosis, which can be assessed by phosphorylated histone H3 [63], [64]. Our observations of a higher mitotic index in ^56^Fe-irradiated samples are indicative of cellular proliferation rather than senescence of the IEC and a number of proliferative signaling factors like NFkB and AP1 are known to be activated by oxidative stress [34]. Persistent proliferative signals in the presence of DNA damage have been projected to propagate mutation in the progeny with survival advantage and carcinogenesis [24], [65]. However, we are yet to have direct evidence to link observed changes in IEC to carcinogenic precursor events. Additional studies will be required to determine the genomic changes as well as to dissect the signaling pathway alterations induced by the persistent sub-lethal oxidative stress in IEC and implicate these changes in the initial events of cellular transformations. However, our results on intestinal tumorigenesis induced by radiation of differing quality in APC^Min/+^ mice, a well-studied mouse model for colorectal cancer, provide supporting evidence of a connection between high-LET radiation induced persistent sub-lethal oxidative stress, mitogenic signals, and carcinogenesis [17].

**Figure 8:**
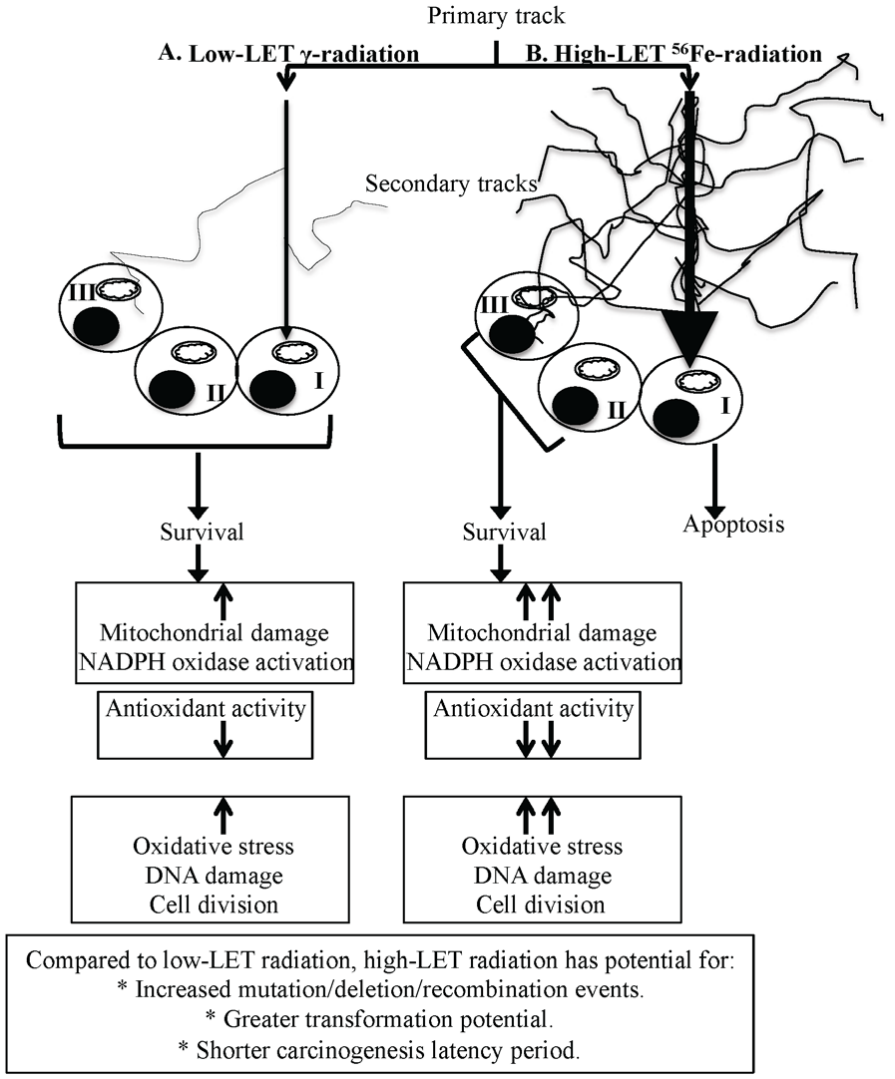
Schematic overview of high-LET radiation-induced persistent oxidative stress. A) Low-LET γ radiation is sparsely ionizing and has very few secondary tracks. B) A primary track of high-LET radiation is densely ionizing and has more secondary tracks than low-LET radiation. I: cell directly hit by a primary track, II: bystander cell, III: cell hit by secondary delta-ray tracks.

Persistent oxidative stress itself has been proposed to propagate into progeny [66]–[68] and because IEC arise from the stem cell compartment of the intestinal crypt-villi system, we propose that the changes in the cellular milieu of the IEC detected in our study are a reflection of the long-term changes in intestinal stem cells. Dynamics of intestinal stem cell compartment, its niche and the progeny in relation to radiation quality dependent oxidative stress remains an important area for investigation in the future to delineate the tissue specific delayed effects of high-LET radiation. Available literature predict that direct traversal by a primary heavy ion particle track due to its high-LET characteristics would most likely result in apoptosis or reproductive death of the stem cell and will not be able to pass on any changes to the progeny [69], [70]. This raises the likelihood that an element of either non-targeted ‘bystander’ effect or targeted secondary delta ray effects is playing an important role in the radiation quality dependent persistent oxidative stress in IEC [24], [55], [71], [72] (**Figure 8**). When considered along with higher CRC in atomic bomb survivors and our previous data in APC^Min/+^ mice showing greater tumorigenesis after ^56^Fe than γ radiation, the results presented here have important implications not only for mechanistic understanding but also for risk estimation of CRC from cosmic radiation exposure in astronauts. Furthermore, our study could facilitate designing strategies to protect GI tissues from cosmic radiation damage during space travel. We also believe that our results could be applied to better understand the long-term consequences of exposure of healthy tissues during heavy ion radiotherapy.

### Conclusions

Oxidative stress has been implicated in gastrointestinal carcinogenesis [73] and our results showing that persistent oxidative stress and oxidative DNA damage in IEC is radiation quality dependent opens the possibility of enhanced cellular transformation with reduced carcinogenesis latency period after high-LET radiation exposure (**Figure 8**). Although we did not observe intestinal tumors during the study timeframe, our observations, we believe, have important implications for understanding long-term consequences of exposure to qualitatively different types of radiation.
